# *Bacillus subtilis *actin-like protein MreB influences the positioning of the replication machinery and requires membrane proteins MreC/D and other actin-like proteins for proper localization

**DOI:** 10.1186/1471-2121-6-10

**Published:** 2005-03-03

**Authors:** Hervé Joël Defeu Soufo, Peter L Graumann

**Affiliations:** 1 Biochemie, Fachbereich Chemie, Hans-Meerwein-Straße, Philipps-Universität Marburg, 35032 Marburg, Germany; 2 Institut für Mikrobiologie, Biologie II, Universität Freiburg, Stefan-Meier-Str. 19, 79104 Freiburg, Germany

## Abstract

**Background:**

Bacterial actin-like proteins have been shown to perform essential functions in several aspects of cellular physiology. They affect cell growth, cell shape, chromosome segregation and polar localization of proteins, and localize as helical filaments underneath the cell membrane. *Bacillus subtilis *MreB and Mbl have been shown to perform dynamic motor like movements within cells, extending along helical tracks in a time scale of few seconds.

**Results:**

In this work, we show that *Bacillus subtilis *MreB has a dual role, both in the formation of rod cell shape, and in chromosome segregation, however, its function in cell shape is distinct from that of MreC. Additionally, MreB is important for the localization of the replication machinery to the cell centre, which becomes aberrant soon after depletion of MreB. 3D image reconstructions suggest that frequently, MreB filaments consist of several discontinuous helical filaments with varying length. The localization of MreB was abnormal in cells with decondensed chromosomes, as well as during depletion of Mbl, MreBH and of the MreC/MreD proteins, which we show localize to the cell membrane. Thus, proper positioning of MreB filaments depends on and is affected by a variety of factors in the cell.

**Conclusion:**

Our data provide genetic and cytological links between MreB and the membrane, as well as with other actin like proteins, and further supports the connection of MreB with the chromosome. The functional dependence on MreB of the localization of the replication machinery suggests that the replisome is not anchored at the cell centre, but is positioned in a dynamic manner.

## Background

Actin provides vital functions as a cytoskeletal component in eukaryotic and in prokaryotic cells. In eukaryotes, actin filaments give mechanical strength to cells in form of a dynamic cytoskeleton, and are structural fibers in muscle contraction. Additionally, actin proteins have motor like functions [1-3], most notably in cell migration through pushing of membranes. Motility receptors turn on WASP family proteins, which binds to and activate the Arp2/3 complex. The latter induces branching and growth of actin filaments [3]. *In vitro*, actin filaments can deform vesicles and thus push membranes, providing the force to elongate cellular extensions such as pseudopods [4,5]. *Listeria monocytogenes *cells move within macrophages through propelling by actin bundles that extend at only one pole of the bacterial cells, due to the ActA protein that is present at one cell pole and that induces rapid polymerisation of actin. Bacterial cells also possess several different actin-like proteins [6]. MreB is essential for cell viability, its depletion leads to a defect in chromosome segregation, and ultimately to the formation of round cells, i.e. to loss of rod cell shape. Except for plasmid encoded ParM protein, which actively partitions plasmids [7], the true mode of action and regulation of bacterial actins is still rather unclear.

During the depletion of *Bacillus subtilis *MreB or of Mbl, the second actin ortholog, or of *Caulobacter crescentus *MreB, origin regions on the chromosomes fail to separate properly, leading to a severe (or in case of Mbl more moderate) segregation defect [8,9], likewise to overproduction of a dominant negative *mreB *allele in *E. coli *[10]. It is unclear, if actin proteins have a direct role, e.g. as an active segregation motor, or an indirect influence on the segregation of chromosomes. In support of an active role, MreB appears to be associated with the nucleoids, in contrast to Mbl [8], which is thought to be involved in the insertion of new cell wall material into the growing peptide glycan layer [11]. Plasmid encoded *E. coli *ParR protein binds to a specific *cis *site on the duplicated plasmids, which are located close to the cell centre, and induces polymerisation of the ParM actin homolog [12]. ParM filaments contain plasmids at their pole ward ends, so two oppositely orientated ParM filaments appear to push plasmids towards each cell pole [7]. On the other hand, *C. crescentus *MreB has been shown to also affect cell shape and the localization of cell wall synthesizing proteins [13], and to play an important role in determining the global polarity of the cell, i.e. by affecting the localization of proteins to the cell pole [9]. MreB and Mbl form helical filaments just underneath the cell membrane [13-16], which in *B. subtilis *are highly dynamic. MreB and Mbl move along helical tracks, with a speed of about 0.1 μm/s, providing potential motor like force [17]. Actin polymerises into a two stranded right handed helix through addition of ATP-bound actin monomers. Actin movement arises through growth at the barbed end of the filament, while actin is released from the pointed end following ATP hydrolysis (a process termed treadmilling). Active pushing is thought to occur through binding of actin monomers to the tip of the filament when the object moves away, thus preventing backward movement, such that the object is driven by Brownian diffusion, with the actin filament dictating a single direction (polymerisation rachett) [1].

Bacterial chromosome segregation is a highly organized process, depending on several essential protein complexes. DNA polymerase localizes to the cell centre throughout most of the cell cycle [18]. During replication, the chromosome moves through this stationary replisome, and duplicated regions are rapidly moved towards opposite cell poles [19], where they are bound and organized by the SMC complex [20]. It is still unknown if the localization of the replisome involves an anchor, or which factors are involved in the central positioning.

We have investigated the role of MreB and Mbl in the positioning of the replication factory, and investigated the role of other actin like proteins and membrane proteins for the localization of MreB. We have found that *B. subtilis *MreC and MreD proteins localize to the cell membrane, and affect the localization of MreB, likewise to Mbl and MreBH, showing that an intricate interplay exists between actin orthologs and MreCD membrane proteins.

## Results

### MreB and MreC have distinct functions in cellular growth and in control of cell shape

**Figure 1:**
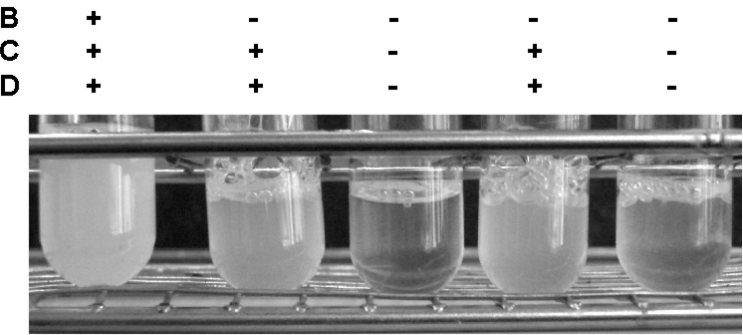
Synthesis of MreB, MreC and MreD is continued or repressed during the exponential growth phase. Depletion of MreB in the presence of MreC and MreD leads to an arrest in growth, compared to cells with continued synthesis of all the proteins, as indicated above the tubes. Depletion of MreB, C and D results in a more rapid cessation of growth.

**Figure 2:**
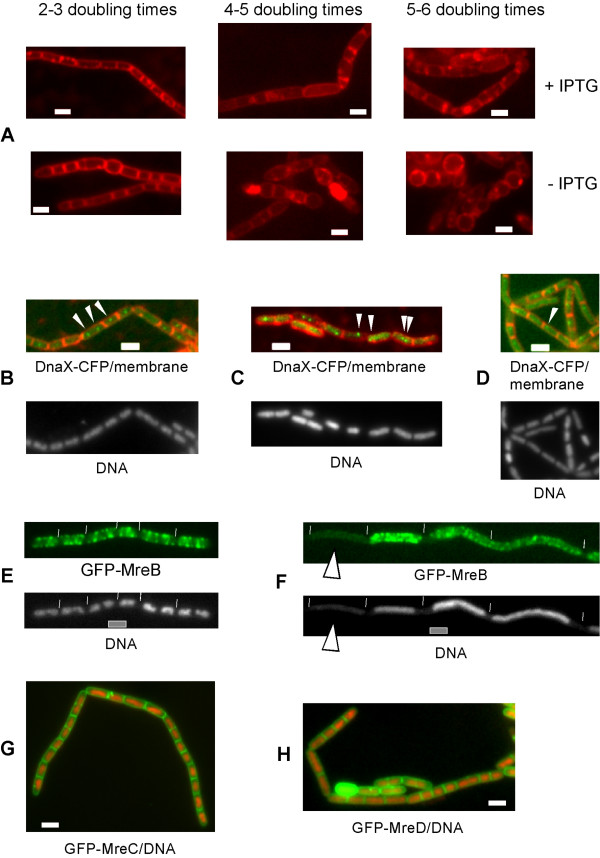
Depletion of MreB in the presence of MreC and of MreD affects both cell shape and segregation of chromosomes, and affects localization of the replication factory. Fluorescence microscopy of exponentially growing *Bacillus subtilis *cells. A) MreB is depleted in the presence (+IPTG, upper panels) or in the absence (-IPTG, lower panels) of MreC and MreD, 2–3, 4–5 and 5–6 doubling times indicate the time after the onset of depletion. B) Localization of DnaX-CFP in wild type cells, or C) 2–3 doubling times after depletion of MreB, or D) 2–3 doubling times after depletion of Mbl. Arrowheads indicate the proper positioning of DnaX-CFP in wild type cells, and its abnormal loacalization during depletion of actin orthologs. E) Localization of GFP-MreB in wild type cells, or F) in *smc *mutant cells, G) localization of GFP-MreC, overlay of GFP-MreC (green) and DNA stain (red), H) localization of MreD, overlay of GFP-MreD (green) and DNA (red). White lines indicate ends of cells, white bars 2 μm.

The depletion of MreB has been shown to lead to a strong defect in chromosome segregation, followed by a loss of rod shape, while the depletion of MreC results in a defect in cell shape, but not in a segregation defect [8,14]. Thus, it is clear that the chromosome segregation defect is due to the lack of MreB. However, repression of transcription of *mreB *may also lead to the depletion of MreC, because the *mreC *gene lies directly downstream of *mreB*, so, it was unknown if depletion of MreB also affects cell shape directly, and if so, to which extent, or if the observed defect in cell shape is due to a polar effect on *mreC*. Since both genes are essential [8,21,22], the lethal defect in cell shape could be caused by the loss of either gene product. To clarify this point, we depleted MreB in the presence of continued synthesis of MreC and MreD (the *mreD *gene lies downstream of *mreC*, and loss of MreD also leads to a defect in cell shape [8]). We introduced a second, IPTG inducible copy of *mreCD *at an ectopic site on the chromosome, which fully complemented the loss of the original *mreCD *genes. Depletion of *mreB *during continued synthesis of *mreCD *from the ectopic site resulted in cessation of growth (Fig. 1, compare first tube with second and fourth), in the formation of round cells (Fig. 2A, upper panels), and in the described defect in chromosome segregation (data not shown). This finding shows that MreB has indeed a dual function, in the formation of rod shaped cells, as well as in chromosome segregation. However, depletion of MreB in the absence of ectopically expressed MreC and MreD led to a considerably different phenotype compared to continued expression of MreCD. In the absence of *de novo *MreCD synthesis, depletion of MreB resulted in a much more rapid growth arrest (Fig. 1, compare second and fourth tubes with third and fifth), cell growth was abolished after about 4–5 doubling times. Moreover, round cells appeared already 2–3 doubling times after the onset of depletion (Fig. 2A, lower left panel), when cells with continued expression of MreCD still had wild type cell morphology [Fig. 2A, upper left panel, cells look indistinguishable from wild type cells (data not shown)]. Additionally, the cell shape was differentially affected during each condition. MreB depleted cells which continued to express MreCD became highly enlarged (Fig. 2A, upper middle and right panel), but were still mostly rod shaped, whereas MreBCD depleted cells became round and entirely lost rod shape (Fig. 2A, lower middle and right panel). Likewise, the depletion of MreC is followed by the rapid formation of small round cells, and an early arrest in growth [8]. Thus, the depletion of MreC has a much more immediate effect on growth compared with MreB, and MreB affects the formation of rod shaped cells in a manner distinct from MreC.

### Depletion of MreB leads to the loss of mid cell localization of the replication machinery

**Figure 3:**
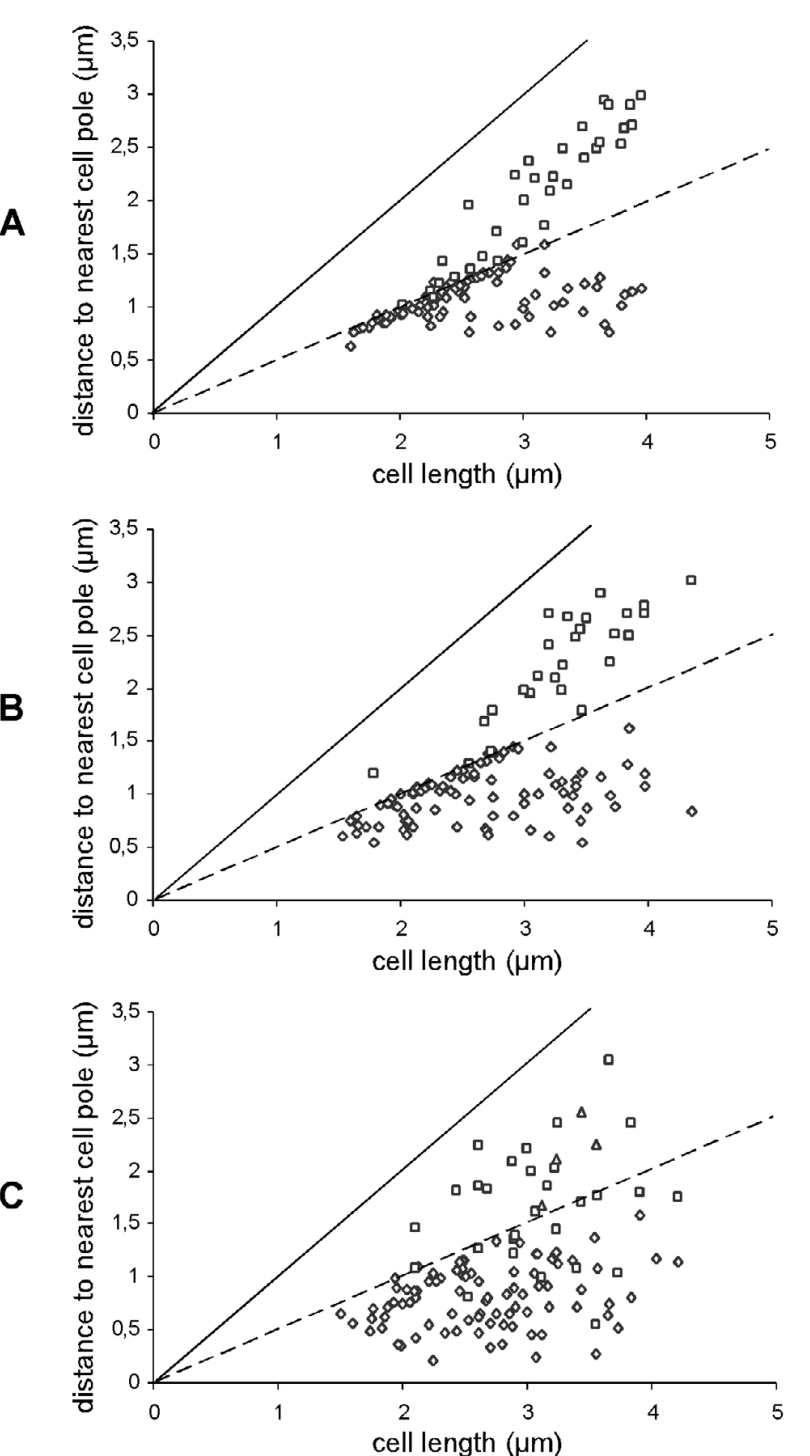
Graphical representation of the position of the replication machinery within wild type or actin-depleted cells. The distance of DnaX-CFP foci to the nearest cell pole was measured and plotted relative to cell size. A) wild type cells, B) cells 2–3 doubling times after depletion of Mbl, C) cells 2–3 doubling times after depletion of MreB. ◇ single focus of focus closest to a pole, □ second focus, Δ third focus.

We wished to further investigate the function of MreB in chromosome partitioning. An important cell biological question is which factors are implicated in the localization of the replication machinery to the cell centre in *B. subtilis *and in *E. coli *cells. To investigate a possible role of actin like proteins in this positioning, we depleted MreB or Mbl in cells expressing DnaX-CFP, the tau subunit of the replication DNA polymerase core machinery. In wild type cells showing clear foci (92%), 67% contained a single focus that was positioned close to the cell centre (< 0.2 μm distance, Fig. 2B, and Fig 3A), 7% had a focus >0,2 μm away from the cell centre, and 26% had two foci that were mostly located around at the cell quarter positions (300 cells have been monitored). The latter cells were the larger cells (> 2.7 to 2.8 μm), as has been described before [23]. Of note, 7% of the double-DnaX-CFP foci were present in smaller cells (<2.7 μm), indicating that the replication forks can also move apart, and come back together (under the growth conditions used, a new round of replication can only occur very late in the cell cycle). Contrarily, 3–4 hours after depletion of MreB, when most cells still retained their rod shape (which is lost after 4–5 doubling times, see above), DnaX-CFP foci were placed at irregular positions within the cells. Only 14% of the cells contained single central foci, while 86% of the foci were off centre (that is more than 0.2 μm away from the cell centre), and were present at random places on the nucleoids (Fig. 2C, Fig. 3C). Additionally, 6% of the MreB-depleted cells contained 3 foci, which was observed in only 1% of the wild type cells, 5% contained two foci within one cell half (never found in wild type cells), and in 3% of the cells, foci were even seen close to a cell pole, which was also never found for wild type cells. However, Fig. 3C shows that in spite of the loss of mid cell positioning, DnaX-CFP foci were still mostly absent from the cell poles, which is due to the fact that there is rarely any DNA at these subcellular places (Fig. 2C). Thus, the replication machinery persists for a long time during depletion of MreB, but is located at random sites on the nucleoids, as illustrated in Fig. 3C.

The depletion of Mbl also had an effect on the positioning of the replication machinery, however, to a much milder extent compared with MreB. 3–4 doubling times after the onset of depletion, 38% of the cells contained a single, mid cell-positioned focus, and 30% two foci in each cell half (roughly at the quarter positions), whereas in 32% of the cells, the DnaX-CFP signals were more than 0.4 μm away from the cell centre (Fig. 2D). Nevertheless, it is apparent from Fig. 3B that although the scatter of DnaX-CFP around the cell centre (and around the quarter positions) is larger in Mbl depleted cells compared with wild type cells, the replication machinery is still largely retained close to the cell centre, contrarily to MreB depleted cells. These results show that directly or indirectly, MreB has a major effect on the positioning of the replisome.

### MreB appears to form several discontinuous helices within each cell

**Figure 4:**
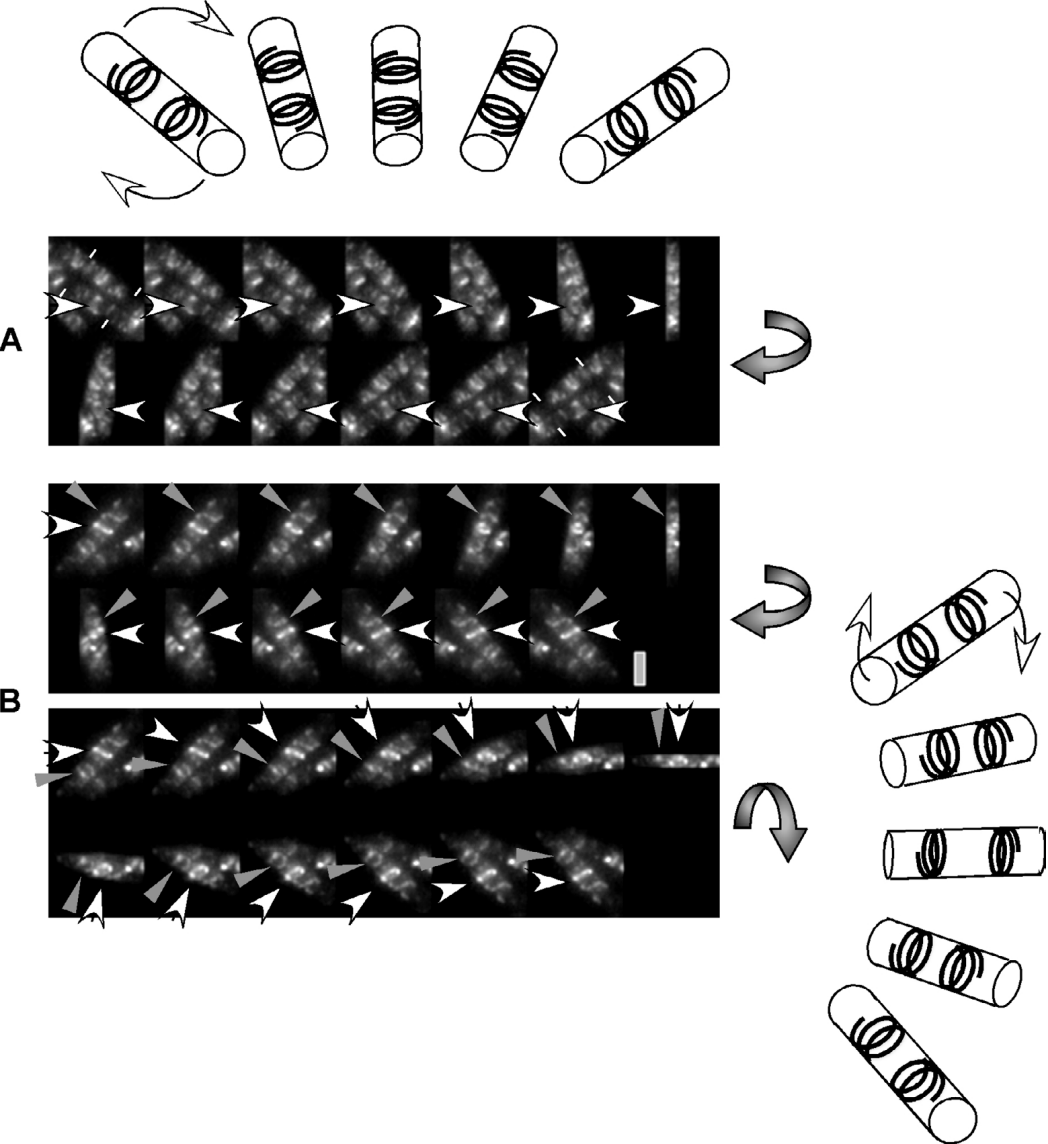
3D reconstruction of stacks of Z sections taken through *B. subtilis *cells expressing MreB-GFP. 180° view of cells (panels are tilted 15° relative to each other as indicated by the grey arrows next to the panels). A) Horizontally turned view of two cells (ends are indicated by white lines, arrow indicates clearly visible helical filament), B) horizontal (upper panel) and vertical (lower panel) view on a single cell (white arrow indicates helical filament, grey arrow half turn filament). The cartoons indicate the rotation, the cartoon on top for the first two panels, the cartoon on the right for the third panel. All images are scaled identically, grey bar 2 μm.

To obtain a more detailed view on the nature of the helical MreB filaments, Z sections were taken through the cells, and 3D image reconstruction was performed on the stacks of fluorescent images. Fig. 4 shows representative reconstructions (cells are turned around 180°, as indicated by the grey arrows, such that the MreB filaments can be seen from 15° angle turns around a 180° view), which clearly show that MreB filaments have a helical path underneath the cell membrane. However, the filaments were not continuous; rather, the cells appeared to contain a variable number of distinct, apparently unconnected filaments. The longest filaments were observed to be only little longer than a full turn around the cell diameter, (indicated by arrowheads in Fig. 4A and 4B), while half turn and much shorter filaments were also present within the cells. Thus, MreB appears to be present as a number of unrelated, membrane-associated very short filamentous structures. However, the reconstructions do not rule out that MreB is organized into longer helices with linkers between the short fragments that are difficult to visualize. It is also apparent from Fig. 4A and 4B, that the fluorescence intensity of the filaments is different within a single cell (compare filaments indicated by arrowheads with other filaments in the respective cell), which was highly reproducible. Thus, MreB helices are heterogeneous within cells, and apparently, do not form cytoskeletal fibres extending continuously throughout the cell. These data are in agreement with our finding, that several MreB filaments or bundles of filaments rapidly move along helical tracks [17], and support our findings that these filaments form independent dynamic structures.

### The localization of MreB is affected by the state of the nucleoids

It has been shown that MreB is closely associated with DNA, because no helical filaments are visible in anucleate cells, in contrast to Mbl or MreBH filaments that are found in anucleate cells [17]. However, MreB filaments were present in cells containing nucleoids during depletion of Topo IV, which leads to a block in full separation of the chromosomes. To investigate, if MreB filaments might be affected by the shape of the nucleoids, we moved the GFP-MreB fusion into *spo0J *mutant cells, which have slightly decondensed DNA, or into *smc *mutant cells, in which the nucleoids are highly decondensed, and which contain less negatively supercoiled DNA compared to wild type cells [24]. Wild type cells contained different numbers of distinct MreB filaments at cellular positions that also contained DNA, but not close to the cell poles, which are devoid of DNA (Fig. 2E). Contrarily, MreB formed somewhat abnormal long filaments in *spo0J *mutant cells (data not shown), and highly aberrant elongated filaments throughout *smc *mutant cells (Fig. 2F), that is the filaments extended right to the cell poles, had fewer gaps than in wild type cells, and the spacing between individual turns was much shorter compared with wild type cells. These findings indicate that the formation of proper MreB filaments is influenced by the state of the chromosomes. In agreement with earlier results, GFP-MreB filaments were not observed in all of the 35 anucleate *smc *mutant cells monitored (forming about 15% anucleate cells [25]).

### Formation of MreB filaments is influenced by MreC and MreD membrane proteins, and by other actin proteins

*MreB *is upstream of *mreC *and *mreD *genes, whose depletion leads to formation of round cells (see above, [8,22,26]). Both gene products are highly hydrophobic, and MreD is predicted to form at least 5 membrane spanning helices (data not shown). N-terminal GFP fusions to both proteins were fully functional, and showed a uniform staining of the cell membrane (Fig. 2G and 2H). Thus, both proteins are associated with the cell membrane. Lee and Stewart have used immuno-gold labelling to show that MreC is predominantly found at the septum between cells [22]. It is clear from Fig. 2G and 2H that MreC and MreD fluorescence is highest at the septum, because two membranes are closely adjacent to each other, which is most likely the explanation for why immuno-gold labels were enriched at this location.

**Figure 5:**
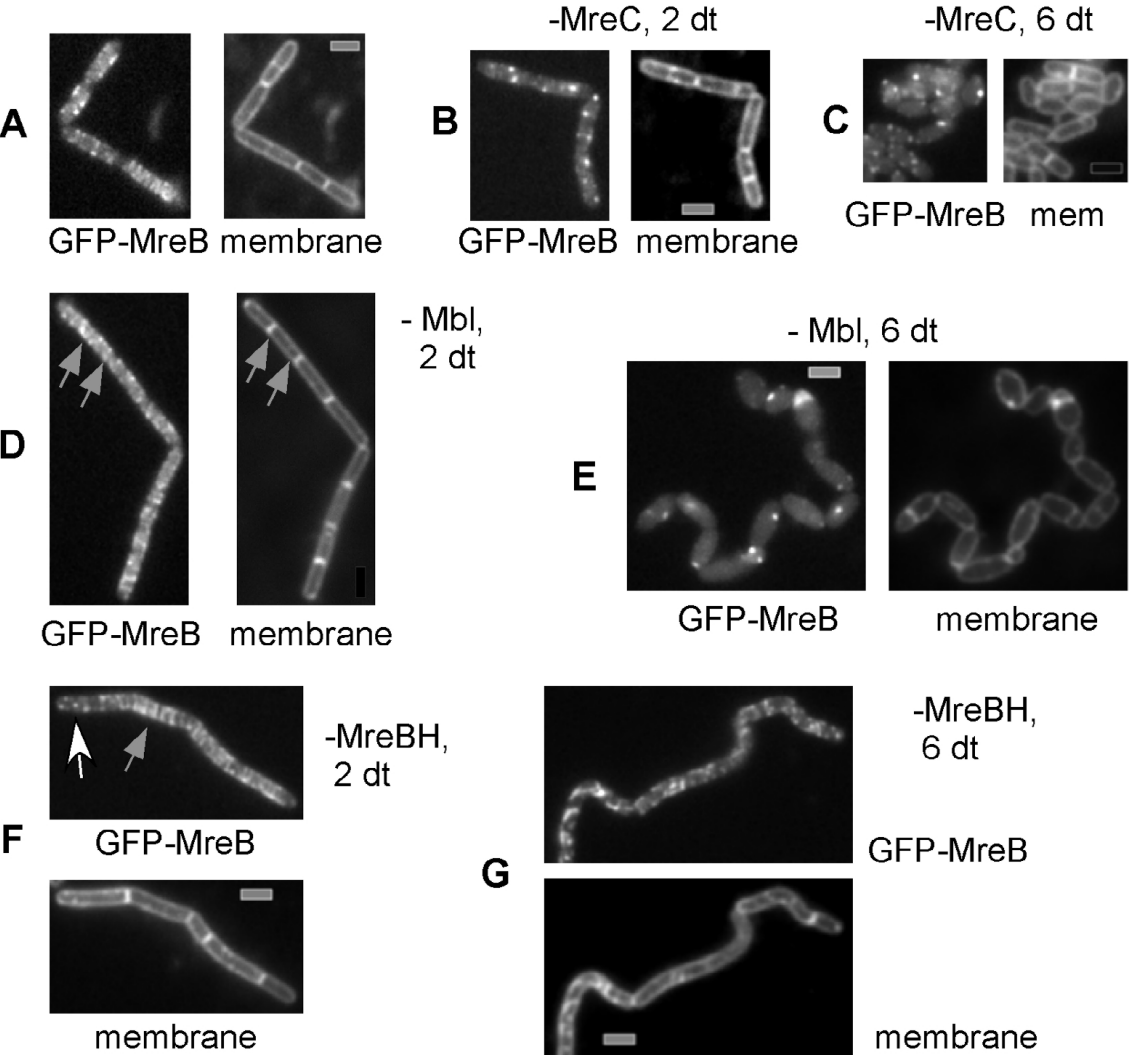
Fluorescence microscopy of *Bacillus subtilis *cells expressing GFP-MreB from an ectopic site on the chromosome. A) wild type cells (helical filaments), B) 2 or C) 4 doubling times after depletion of MreC (loss of filaments), D) 2 or E) 6 doubling times after depletion of Mbl (abnormal filaments and later loss of filaments), F) 2 or G) 6 doubling times after depletion of MreBH (abnormal filaments). Grey arrows point out extended GFP-MreB filaments, and the white arrow indicates GFP-MreB foci. Grey bars 2 μm.

We wished to investigate if formation of helical filaments of MreB depends on the other two actin proteins, or on MreC and MreD, which could provide membrane association of the helical filaments. We moved a *gfp-mreB *copy to the amylase locus under control of the hyperspank promoter that is induced by IPTG, while *mbl*, *mreBH*, *mreC *or *mreD *genes were driven by the xylose promoter that is induced by xylose (in fructose medium), and is repressed in glucose medium lacking xylose. GFP-MreB filaments were observed in 85–90% of exponentially growing cells in the presence of IPTG (Fig. 5A). After 1–2 generation times of growth of *pxyl-mreC *cells in the absence of xylose, 65% of the cells contained GFP-MreB foci, rather than filaments, and only 20% of the cells showed GFP-MreB filaments, while the cell morphology was still normal (Fig. 5B). When cells started to become round and ceased to grow after 3–4 generation times, only 15% of the cells showed MreB filaments, and after more than 6 generation times, when most cells had a cocci like morphology, only 5% contained visible GFP-MreB helices, while most cells contained GFP-MreB foci (Fig. 5C). Depletion of MreD led to a similar albeit much less drastic phenotype (data not shown). Thus, MreC and MreD are required for the formation of proper helical filaments of MreB.

As opposed to depletion of MreC or MreD, depletion of Mbl or of MreBH leads to a high number of cells, in which MreB filaments extended throughout the entire cell (about 40% of the cells 2 doubling times after depletion of Mbl or of MreBH, indicated by grey arrows, Fig. 5D and 5F), or in which only foci were visible (25%, white arrow, Fig. 5F). In Mbl depleted cells (which are bulgy and twisted [8,14,27]), only highly aberrant and weak GFP-MreB filaments were detectable (Fig. 5E), while the more vibrio-shaped MreBH depleted cells contained highly irregular MreB filaments (Fig. 5G). Thus, formation of proper MreB filaments is affected by Mbl and MreBH. However, even the highly abnormal MreB filaments in Mbl depleted cells are able to support cell viability, albeit at a highly reduced level (*mbl *deleted cells grow extremely slowly [14,27]).

## Discussion

This work provides several important conclusions on the function and localization of the *B. subtilis *actin ortholog MreB. Our experiments establish that MreB has a dual function, it is vital for the formation of proper rod shape of the cells, and for regular chromosome segregation. However, its function in cell shape is different from that of MreC, or of MreD. The depletion of MreC and MreD leads to rapid cessation of growth and to the formation of small round cells, whereas the sole depletion of MreB results in the formation of large oval shaped cells, and a slower occurring growth arrest. Interestingly, though, we found a connection between MreC and MreB, because during depletion of MreCD, MreB formed fewer and usually abnormally shaped helical filaments. Similar observations have recently been made in *E. coli *cells [28]. Our experiments show that MreC and MreD localize throughout the *B. subtilis *membrane, establishing a link between MreB and the membrane. We speculate that MreC and MreD might provide low affinity binding sites for MreB, such that the filaments extend underneath the membrane in a regular helical pattern. Our results also suggest a dual function for MreC, because its deletion affects the localization of MreB (which is apparently not severe enough to strongly interfere with chromosome segregation), as well as cell shape (in a manner distinct from MreB).

An important, if not crucial function of MreB is the positioning of the replication machinery in *B. subtilis *cells. Soon after the depletion of MreB, the replisome lost its central position in the cell, before a change in cell shape was apparent. The depletion of Mbl had only a minor effect on the localization of the replisome, showing that MreB also affects the positioning of an intracellular protein assembly. Our results do not distinguish between the possibilities that the lack of MreB activity results in the loss of central localization of the replisome, which in turn leads to a segregation defect, or that a more direct defect in chromosome segregation due to the lack of MreB might cause mislocalization of the replisome. However, it is tempting to speculate that MreB could actively push DNA away from the central replisome towards opposite cell poles, and that the net result of this simultaneous pushing of ejected DNA towards opposite directions might lead to a balanced positioning of the replisome towards the cell centre, without any need for an anchor. This is in agreement with recent data showing that the replication machinery is highly mobile around the cell centre [29,30].

An intriguing property of bacterial actin orthologs is the formation of highly dynamic helical filaments underneath the cell membrane that for some members of this protein family are thought to extend through the entire cell length [14,16]. Three dimensional image reconstructions have helped to resolve the nature of the helical MreB filaments in live cells. MreB does not form a closed cytoskeleton like structure, but different forms of filaments within a single cell. These filaments can stretch along a half turn up to a full turn underneath the membrane, but are not clearly connected with each other. This is in agreement with findings showing that several MreB filaments move continuously along helical tracks [31], generating motor-like intracellular movement.

We also provide evidence that the formation of MreB filaments is affected by the nature of the nucleoids, and by the other actin like proteins. In *smc *mutant cells, MreB filaments are abnormally spaced and extended, and to a much lesser extent in *spo0J *mutant cells. This further supports and extends our earlier findings that a connection exists between MreB and the nucleoids. Interestingly, *smc *mutant cells are elongated and frequently twisted and wider than wild type cells [32], which might be due to the effect on MreB. Likewise, the depletion of Mbl or of MreBH interfered with formation of proper MreB filaments, revealing a tight link between the three actin orthologs. It will be interesting to investigate how these proteins localize relative to each other within a single cell, and if they even physically interact with each other.

## Conclusion

Our findings show that an intricate interplay exists between MreB, membrane associated MreC and MreD proteins, other actin orthologs, the replication machinery and the nucleoids, shedding light on the question why the depletion of MreB affects both, chromosome segregation and cell shape. What remains to be investigated are several important questions, e.g. what is the mode of interaction between MreB and the MreCD proteins or with Mbl and MreBH, and to identify the link between MreB and the nucleoids or the replisome, to distinguish between the causality of defects caused by the loss of MreB activity. Also, it will be highly revealing to identify the possible load MreB might be pushing, if its dynamic movement indeed constitutes a motor function within the prokaryotic cell.

## Methods

### Growth conditions

*Escherichia coli *XL1-Blue (Stratagene) or *B. subtilis *strains were grown in Luria-Bertani (LB) rich medium supplemented with 50 μg/ml ampicillin or other antibiotics, where appropriate. For induction of the hyperspank promoter, the culture media were supplemented with 0.1 to 1 mM isopropyl-β-D-thiogalactopyranoside (IPTG). For induction of xylose promotor, glucose in S7_50 _medium was exchanged for 0.5% fructose and xylose was added up to 0.5%.

### Constructions of plasmids

*Gfp mut1 *including MCS was amplified from pSG1729 [33] and was cloned into pSG1164 [33] in which the *gfp mut1 *for C-terminal fusion had been excised using *Kpn*I and *Spe*I. The resulting plasmid pHJDS1 was used to generate N-terminal GFP fusions at the original gene locus. To obtain inducible N-terminal GFP fusion alleles of *mreC *or of *mreD *at the original locus, the 5' prime regions (350 to 500 bp) of the genes were PCR amplified and inserted in the *EcoR*I and *Apa*I sites of plasmid pHJDS1 to generate pJS14 or pJS15, respectively. To create a fusion of GFP to the N-terminus of *mreB*, *mreC or mreD *at an ectopic site on the chromosome, the entire sequences of theses genes were PCR amplified and inserted into the *Eco*RI and *Apa*I sites of plasmid pSG1729 [33] to generate pJS17, pJS19, or pJS20, respectively. To generate an IPTG inducible copy of *gfp-mreB *(pJS22) or of *mreCD *(pJS23) at the amylase locus, *gfp-mreB *was PCR amplified from pJS17 and *mreCD *from *B. subtilis *PY79 chromosomal DNA, and the products were cloned as *Hin*dIII-*Sph*I or as *Sal*I-*Sph*I fragments, respectively, immediately downstream of the hyperspank promotor in plasmid pDR111 (kind gift of D. Rudner, Harvard Medical School).

### Bacterial strains

To express GFP-MreC or GFP-MreD at their original locus in *Bacillus*, pJS14 or pJS15 plasmids were transformed into wild type *B. subtilis *(PY79) selecting for chloramphenicol resistance (Cm, 5 μg/ml) to generate strains JS14 (*Pxyl-gfp-mre*C) or JS15 (*Pxyl-gfp-mreD*), respectively. For GFP N-terminal fusions at the *amy *locus, plasmids pJS19 and pJS20 for *mreC and mreD *were transformed into PY79 selecting for spectinomycin resistance (spec, 25 μg/ml) to generate strains JS19 (*Pxyl-gfp-mreC::amy*) and JS20 *(Pxyl-gfp-mreD::amy*), respectively. Strain JS32, in which *mreB *can be depleted in the presence or absence of *mreCD*, was created by transforming compentent JS1 cells with chromosomal DNA of JS32. To examine the subcellular localization of GFP-MreB in *spo0J *or in *smc *null cells, strain JS19 was transformed with chromosomal DNA from strains AG1468 [34] or PGΔ388 [25], generating strains JS23 (*Pxyl-gfp-mreB::amy, ΔspoOJ*) and JS24 (*Pxy-gfp-mreB::amy, smc::kan*) respectively. To be able to visualize the localisation patterns of labelled MreB helices in cells depleted of MreC, MreD, Mbl and MreBH cells, chromosomal DNA from strains JS3 (*Pxyl-mreC*), JS4 (*Pxyl-mreD*), JS2 (*Pxyl-mbl*) and JS5 (*Pxyl-mreBH*) was used to transform strain JS25 (*Phyperspank-gfp-mreB::amy*)selecting for Cm and spec, generating strains JS29 (*Phyperspank-gfp-mreB::amy, Pxyl-mreC*), JS30 (*Phypespank-gfp-mreB::amy, Pxyl-mreD*), JS28 (*Phyperspank-gfp-mreB::amy, Pxyl-mbl*), and JS31 (*Phyperspank-gfp-mreB::amy, Pxyl-mreBH*). To express DnaX-CFP in MreB or Mbl depleted cells, chromosomal DNA from JS1 and JS2 was used to transform PG24 competent cells.

### Image acquisition

For microscopic analysis, *Bacillus *strains were grown in S7_50 _defined medium [35] complemented with 1% casamino acids. Fluorescence microscopy was performed on an Olympus AX70 microscope. Cells were mounted on agarose gel pads containing S7_50 _growth medium on object slides. Images were acquired with a digital CCD camera; signal intensities and cell length were measured using the Metamorph 4.6 program (Universal Imaging Corp., USA). For and 3D reconstruction, 10 to 12 images (spacing between 0.2 to 0.38 μm) were taken through the focal plane, and processed in Metamorph 6 program. DNA was stained with 4',6-diamidino- 2-phenylindole (DAPI; final concentration 0.2 ng/ml) and membranes were stained with FM4-64 (final concentration 1 nM).

## Authors' contributions

H J D S performed all experiments, PLG helped with 3D image reconstructions, conceived the study, and participated in its design and coordination. All authors read and approved the final manuscript.
